# Exploring dog saliva as a non-invasive alternative to blood sampling for chemical exposome studies: analysis of synthetic phenolic antioxidants and PFAS

**DOI:** 10.3389/fvets.2025.1734889

**Published:** 2026-02-02

**Authors:** Jana M. Weiss, Josefin Engelhardt, Lorena Franco Martínez, Asta Tvarijonaviciute

**Affiliations:** 1 Department of Environmental Science, Stockholm University, Stockholm, Sweden; 2 Clinic for Internal Diseases, Faculty of Veterinary Medicine, University of Zagreb, Zagreb, Croatia; 3 Interdisciplinary Laboratory of Clinical Analysis, Interlab-UMU, Regional Campus of International Excellence "Campus Mare Nostrum", University of Murcia, Murcia, Spain

**Keywords:** 2,4-DBP, BHA (butylated hydroxyanisole), BHT (butylated hydroxytoluene), blood serum, Dogs, PFAS (per- and polyfluoroalkyl substances), saliva, synthetic phenolic antioxidants (SPAs)

## Abstract

**Background:**

Humans and companion animals are continually exposed to mixtures of synthetic chemicals from household products, textiles, food, and personal care items. Epidemiological studies of emerging contaminants typically rely on invasive blood sampling, whereas saliva presents a non-invasive and cost-effective alternative matrix that reflects both local and systemic physiological changes. In this pilot study, paired dog saliva and blood serum were analysed for two chemical groups of concern, that is, synthetic phenolic antioxidants (SPAs) and per- and polyfluoroalkyl substances (PFAS), to investigate whether saliva can be used for chemical exposome studies.

**Methods:**

Blood serum and saliva samples of a total of 30 dogs were included in this study. All dogs were privately owned, representing different breeds, and were presented to private veterinary clinics in the Murcia Region, Spain. Samples were analysed using acetonitrile for denaturation and extraction, and clean-up using enhanced matrix removal powder. The extracts were analysed using complementary liquid and gas chromatography coupled with mass spectrometers. Dogs were divided into three groups (*n* = 10 per group): healthy normal-weight dogs, healthy obese dogs, and dogs diagnosed with mammary tumours.

**Results:**

Fourteen SPAs (including five metabolites) and eight PFAS were quantified in the dog’s saliva and/or serum. Generally, the levels were higher in serum than in saliva. None of the dogs were free of contaminants. The SPA levels were dominated by the 2,4-di-tert-butylphenol (2,4-DBP), found in 93% of saliva and 100% of serum samples, ranging from <LOQ-860 ng/g in saliva and 130–2,100 ng/g in serum. The levels of PFAS were dominated by perfluorononanoic acid (PFNA) and perfluorosulfonic acid (PFOS), which were quantified in more than 70% of the samples. No correlation between levels and the three groups, or the paired serum and saliva samples could be established.

**Conclusion:**

This study confirmed that SPAs and PFAS can be found in the saliva of dogs. The levels of PFAS in dog’s serum were similar to those generally reported in human blood. Furthermore, this study confirms that dogs are exposed to SPAs at concerningly high levels, given the limited knowledge regarding their toxicity.

## 1 Introduction

Humans and companion animals are exposed to a wide range of synthetic chemicals through their surrounding environment. These chemicals are used, for example, in household products, textiles, and building materials, and are added to the food and personal care products. Exposure to the chemicals can occur directly while using the products or indirectly when the chemicals enter the environment through waste and garbage disposal, subsequently entering the food chain and drinking water. Companion animals and humans are exposed to the same mixture of chemicals originating from the indoor environment (1–5). Consequently, companion animals such as dogs have been suggested as model organisms for human exposure and health effects, as several non-communicable disorders are shared between the species. The lack of understanding the consequences of the chemical exposome is one of the nine planetary boundaries (novel entities), and the safe operating space is considered transgressed (6).

Synthetic phenolic antioxidants (SPAs) are tert-butylated phenolic compounds with antioxidative properties that are widely used in cosmetics, preservatives, food-contact materials (FCMs), plastics and rubber, glues, and adhesives (ECHA Chemical Database). Recently, concerning levels of SPAs have been analysed in human blood (7, 8) and urine (9) of adults, and exposure has also been reported to the fetus and children (10–13). However, to the best of the authors’ knowledge, comparable studies have not been conducted in dogs. Nevertheless, human and dog exposure to SPAs can occur from ingestion of indoor dust (14), via dermal application of personal care products (15), via food that has been contaminated with FCM containing SPAs (16), or from intentional addition to food and feed as conservatives. In Europe, butylated hydroxytoluene (BHT) and butylated hydroxyanisole (BHA) can be added to animal feed at concentrations of up to 150 mg/kg of complete feed (EC Food and Feed Information Portal Database) (17).

Perfluoroalkyl and polyfluoroalkyl substances (PFAS) are synthetic chemicals extensively utilised for their uniquely useful chemical and physical properties. Since the 1950s, these chemicals have been extensively used in industrial, commercial, and consumer products, including non-stick cookware, textiles, firefighting foams, food packaging, and much more (18). These substances are pervasive in the environment and persist in air, water, soil, dust, and food, leading to widespread exposure among humans and animals (19, 20). Serum PFAS levels in companion animals often mirror those found in their human owners, suggesting shared environmental exposure pathways (2, 3, 21). Toxicologically, PFAS exposure has been linked to several adverse health outcomes. In humans, these outcomes include increased cholesterol levels, liver damage, immune suppression, and endocrine disruption (22). Similarly, in companion animals, PFAS exposure has been associated with alterations in liver enzymes, cholesterol levels, and thyroid hormones (19).

Currently, epidemiological studies focusing on the risks associated with exposure to novel and emerging chemicals commonly utilise blood samples. Nevertheless, the process of blood sampling is both physically invasive and technically challenging. In contrast, the use of saliva represents a reduction in the stress associated with the sampling process due to its non-invasive nature and a decrease in costs due to the absence of the need for a specialized workforce. Furthermore, in recent years, there has been a significant shift in the perception of saliva within the field of biological sciences. It has been demonstrated that saliva is not merely a digestive fluid (23) but contains a diverse array of molecular and bacterial compounds that can be altered in the context of local and systemic pathologies (24).

In this pilot study, SPAs (including metabolites) and PFAS were analysed in paired dog saliva and blood serum, using complementary gas and liquid chromatography coupled to mass spectrometry (GC–MS and LC–MS). The primary aims were to (a) evaluate whether saliva can be used for chemical exposome studies and (b) to determine the dog’s exposure to PFAS and SPAs. In addition, the limited dataset was statistically evaluated to assess the correlation between analytes in both matrices and to explore differences between the three groups of dogs with different health status.

## 2 Materials and methods

### 2.1 Samples

For this study, surplus samples from a previous research project were employed. Blood samples were collected for reasons unrelated to this study, due to the decision of the veterinary specialist in charge of the animal. In this study, only the serum remaining after biochemical analysis was used.

Paired blood serum and saliva samples of a total of 30 (9 male and 21 female) dogs with a mean age of 9.2 years (SD 3.7 years) were included in the present study (Supplementary Table S1). All animals were privately owned dogs of various breeds presented to private veterinary clinics of the Murcia Region, Spain. Based on clinical evaluation and body condition score, animals were divided into three groups (*n* = 10 per group): healthy normal-weight (HN), healthy obese (HO), and dogs with mammary tumours (MT).

In all the cases, samples were collected after at least 8 h of fasting. Saliva was collected using a previously reported non-invasive method (24). For saliva sampling, a piece of sponge was placed in the dog’s mouth for 1–2 min and then passed into a Salivette device (Salivette®, Sarstedt AG & Co., Nümbrecht, Germany) for centrifugation (3,000 × g, 10 min, 4 °C), and saliva supernatants were transferred to 1.5 mL polypropylene tubes. Saliva sampling blanks were prepared by sampling a single anonymous individual using a sponge and by collecting saliva via direct drooling into the polypropylene tubes; both procedures were performed in triplicate. Blood samples were collected by the venipuncture of the jugular or cephalic vein in tubes containing a coagulation activator and a gel separator (Tapval, Aquisel, Spain) and maintained at room temperature (25 °C) until visible clot reaction. Samples were centrifuged (3,500 × g, 5 min, 4 °C), and serum was transferred to 1.5 mL polypropylene tubes. Samples were stored at −80 °C and shipped on dry ice until analysis at the Department of Environmental Science, Stockholm University, Sweden.

Sample collection and usage were reviewed and approved by the Animal Experimentation Ethics Committee (CEEA) of the University of Murcia and the General Directorate of Livestock, Fisheries and Aquaculture of the Region of Murcia, Spain, under approval number A13170503, in accordance with the European Council Directives on the protection of animals used for scientific purposes. In addition, the study was conducted in compliance with the ARRIVE (Animal Research: Reporting *of In Vivo* Experiments) guidelines for animal care and use.

### 2.2 Chemical analysis

The analytical method was based on a previously published method for the determination of phenolic compounds in human blood (7), which describes the analysis of both the conjugated and the free fraction of phenolic analytes in 1 mL of serum. As the volume of serum and saliva samples from the dogs was limited, the method needed some adaptations. First, only the free fraction of SPAs and PFAS was determined in this study. Second, ca 200 μL of serum or saliva were analysed, and the volume of solvents and chemicals was reduced (no re-extractions) to minimise background contamination. Traditionally, PFAS analysis is carried out using polypropylene tubes to avoid glass absorption. In this study, we used glass tubes to prevent SPA contamination from plastic material. Much effort was spent on minimising background contamination due to the widespread use of these chemicals. The glassware was burned at 300 °C for 24 h, the solvent was distilled, and the clean-up matrix was washed.

Details regarding chemical information, analytical standards, instrumental settings can be found in Engelhardt et al. (7) for SPA analysis, and in Engelhardt et al. (25) for PFAS analysis.

*Sample preparation:* In detail, to ca 200 μL sample (serum or saliva), 1 ng of the internal standards were added (Supplementary Table S2) and left overnight to equilibrate with the sample. The saliva volume available was, in some cases low, ranging from 64 μL to 210 μL. Two mL of distilled acetonitrile (ACN, HiPerSolv Chromanorm for HPLC, VWR International bv, Leuven, Belgium) was added to the sample for denaturation and extraction. The sample was vortexed, cradled for 5 min, and centrifuged at 3,500 relative centrifugal force (rcf) for 5 min at 16 °C. The supernatant was transferred to a new glass tube containing 50 mg of prewashed (2 times with 1 mL distilled ACN) Enhanced Matrix Removal-Lipid powder (Bond elut EMR-Lipid, Agilent Technologies, Santa Clara, United States). The tube was then vortexed for 10 s and centrifuged at 3500 rcf for 5 min at 16 °C. The supernatant was transferred to a new glass tube, and 200 mg anhydrous magnesium sulphate (MgSO_4_) was added to the extract to remove any remaining water, vortexed, and centrifuged at 3500 rcf for 5 min at 16 °C. The supernatant was transferred to a new glass tube and evaporated with a gentle stream of nitrogen gas to ca 100 μL. The volumetric standards M8PFOS (1 ng) and CB207 (0.5 ng) were added for the quantification of the recovery of the internal standards using LC–MS and GC–MS analysis, respectively. The sample extract was stored in the freezer until analysis.

As quality control (QC) samples, method blanks (*n* = 7), saliva sampling blanks (*n* = 3 + 3), and certified reference material (CRM, human serum NIST 1957, *n* = 3) were analysed. The method blank sample matrix was prepared by adding a drop of previously analysed olive oil to 10 mL distilled ACN. Background contamination from saliva sampling was assessed by subtracting the analyte concentrations found in the drool samples from those found in the sponge-collected samples.

*Instrumental analysis:* Short, the sample extract was first injected (1 μL) on a Trace 1,300 gas chromatograph coupled to a triple quadrupole mass spectrometer (GC-TSQ 9000, Thermo Fisher Scientific Inc.). The injection temperature was 280 °C, and extracts were injected in a splitless mode. The chromatographic separation was achieved using a DB-35MS UI column (30 m, diameter 0.25 mm with a 0.25 μm film, Agilent J&W GC columns Agilent Technologies) with helium as carrier gas. The temperature programme was 90 °C (1.5 min hold), 10 °C/min up to 310 °C (5.5 min hold). The ionization mode was set to positive electron ionization in multiple reaction monitoring mode using timed events [see details in (7)].

The matrix-matched calibration curves were used to quantify SPA analytes on the GC-TSQ, utilising commercially obtained charcoal-stripped fetal bovine serum (Merck), as matrix effect could be observed for the analytes (Supplementary Table S3). The SPA concentration of the calibration curve ranged from 0.03 to 300 ng/g using 10 points, with eight points distributed in the lower concentrations. The two high calibration points were added to cover the concentration of some analytes with high concentrations. The calibration curves were accepted if the linearity criteria *r*^2^ > 0.975 were met.

The same extract as analysed on the GC-TSQ was further analysed using a Dionex UltiMate™ 3,000 ultra-high performance liquid chromatography combined with a Q Exactive™ HF hybrid Quadrupole-Orbitrap™ mass spectrometer (LC-Orbitrap, Thermo Fisher Scientific Inc.). Background contamination from the mobile phases was delayed using an isolator column XBridge™ C18 (2.1 × 50 mm, 3.5 μm particle size, Waters), mounted before the injector. The injection volume was 5 μL, and the flow rate of the mobile phases was set to 0.4 mL/min. A BEH C18 column (2.1 × 50 mm, 1.7 μm particle size, Waters) and a guard column BEH C18 (2.1 × 5 mm, 1.7 μm particle size, Waters) were used for chromatographic separation. For PFAS analysis, an 8-point calibration curve was used with a concentration span of 0.02–15 ng/g.

The mobile gradient started with 90% A (2 mmol/L ammonium acetate in 95% water and 5% ACN) and 10% B (2 mmol/L ammonium acetate in 99% ACN and 1% water), 0.5 min of hold time, mobile phase B increased to 100% during 7.5 min, 3 min of hold time and then switched back to 90% A to equilibrate the column. The analysis was run in negative ionization mode using full scan and data-dependent acquisition with an inclusion list of more than 30 target analytes and 40 suspect features [see details in (7)]. The peak integration was performed using Tracefinder (version 4.1, Thermo Fisher Scientific Inc).

Only PFAS and SPAs detected in dog saliva or serum are reported here. The instrumental limit of quantification (LOQ) was determined as the lowest calibration point with a bell-shaped peak (Supplementary Tables S3, S4). The method LOQ (MLOQ) and the sampling LOQ (SLOQ) were calculated as the average blank level plus one standard deviation (Supplementary Tables S3, S4). The saliva sampling was tested for background contamination, but not the serum sampling. If the analyte was present and stable in the blank (RSD < 30%), the average blank was subtracted from the reported levels.

### 2.3 Statistical analysis

The levels are reported based on weight to be able to compare with previously reported levels. Statistical evaluation was conducted on analytes with a quantification frequency (QF) of at least 30% of the samples in the two matrices. For statistical evaluation and creation of the figures, results below LOQ were set to LOQ/2, and non-detects (n.d.) to LOQ/10.

All statistical analyses were performed using R v3.2.2 (R Core Team, 2013). Data normality was assessed using both the Shapiro–Wilk test and visual inspection of Q–Q plots. Given the sample size and the Shapiro–Wilk results (*p* < 0.05), all variables were considered non-normally distributed. Therefore, non-parametric tests were applied.

Correlations between paired serum and saliva were evaluated using Spearman’s rank correlation test. Comparisons among analytes and the three health groups were conducted using the Kruskal–Wallis test, followed by Dunn’s *post hoc* test. To reduce the risk of type II error in the context of limited sample size, post hoc testing was also performed for variables with Kruskal–Wallis *p* ≤ 0.3. Categorical variables (i.e., sex) were analysed in relation to serum and saliva analytes using the Mann–Whitney *U* test. In all analyses, *p* < 0.05 were considered statistically significant.

## 3 Results

### 3.1 Analytical performance

In this pilot study, a previously established method developed for the analysis of a wide range of synthetic phenolic compounds in blood serum was used, with minor adaptations. No recovery study on neither saliva nor blood serum was performed here, but previously established recoveries of SPAs in blood serum are reported in Supplementary Table S3. The recoveries of the internal standards DBP-d21 and the labelled PFAS were similar in blood serum and saliva (Supplementary Table S2). The CRM sample (NIST 1957) was analysed in triplicates and compared to previously reported SPA levels reported in the same material (Supplementary Table S3) (7). The agreement was found between 84 and 220%, except for 2,6-di-tert-butyl-4-(hydroxymethyl)phenol (BHT-OH). BHT-OH in this study was 10 times higher than that reported earlier. Although, in the previous study, 2.7 ng/g serum of BHT-OH was found in the conjugated fraction but not analysed here. The repeatability was measured by the relative standard deviation (RSD) of the CRM sample analysed in triplicates, which was good (below 12%).

The recoveries of the PFAS internal standards were satisfactory (Supplementary Table S2). Compared to the levels reported by NIST (26), PFAS in the CRM (NIST 1957) were within 30% deviation (82–127%) on an average (Supplementary Table S3). Based on this finding, it was judged that the applied sample preparation method developed for phenol analyses was also suitable for PFAS analysis.

### 3.2 Exposure

In Table 1 and Supplementary Tables S3, S4 analyte concentrations are reported above the limit of quantification, and levels below are reported with their corresponding LOQ, or as non-detects (n.d.) when no peak was indicated. Fourteen SPAs (including five BHT metabolites) and eight PFAS were determined in dog’s saliva and/or serum. In the dog saliva, 2,4-di-tert-butylphenol (2,4-DBP. <LOQ-860 ng/g saliva), 3,5-di-tert-butyl-4-hydroxybenzaldehyde (BHT-CHO. <LOQ-11 ng/g saliva), and perfluorononanoic acid (PFNA. n.d.-0.82 ng/g saliva) were quantified in >90% of the samples, followed by BHA (n.d.-11 ng/g saliva) and perfluorooctanesulfonic acid (PFOS, linear, and branched; <LOQ-6.2 ng/g) quantified in >70% of the samples (Table 1). 2,4-DBP (130–2,100 ng/g serum), BHA (0.35–12 ng/g serum), 2,6-Bis-(1,1-dimethylethyl)-2,5-cyclohexadiene-1,4-dione (BHT-Q. 19–710 ng/g serum) and PFOS (linear and branched, 1.0–19 ng/g) were quantified in all dog blood serum samples (QF 100%).

**Table 1:** Quantification frequency (QF), average, median, range (minimum and maximum) SPA and PFAS levels quantified in dog saliva and serum (ng/g).

Analyte	Dog saliva (ng/g saliva)				Dog serum (ng/g serum)			
	QF	Average	Median	Range	QF	Average	Median	Range
AO1135	31%	9.3	<LOQ	n.d.-140	48%	11	<LOQ	n.d.-87
AO2246	0%				14%	2.5	<LOQ	n.d.-40
AO22E46	0%				14%	0.06	<LOQ	n.d.-0.57
AO4426	3%	0.02	<LOQ	n.d.-0.35	76%	3.0	1.4	n.d.-21
AO44B25	0%				14%	9.4	<LOQ	n.d.-125
2,4-DBP	93%	260	200	<LOQ-860	100%	590	340	130–2,100
AO246	0%				67%	0.047	0.05	n.d.-0.094
BHA	72%	2.3	1.9	n.d.-11	100%	2.7	1.8	0.35–12
BHT	0%				14%	1.8	1.1	<LOQ-9.9
BHT-CHO	90%	4.0	3.3	<LOQ-11	95%	11	6.6	<LOQ-55
BHT-COOH	45%	8.6	<LOQ	n.d.-100	71%	17	7.9	n.d.-120
BHT-OH	38%	0.31	<LOQ	n.d.-1.5	38%	4.5	<LOQ	n.d.-26
BHT-Q	34%	8.0	<LOQ	<LOQ-46	100%	140	88	19–710
BHT-quinol	28%	2.4	<LOQ	<LOQ-22	90%	3.5	2.3	<LOQ-12
PFOA	0%				77%	0.39	0.32	n.d.-1.1
PFNA	93%	0.16	0.094	n.d.-0.82	87%	0.57	0.42	n.d.-3.7
PFDA	0%				80%	0.59	0.48	n.d.-1.5
PFUnDA	0%				47%	0.24	0.09	n.d.-1.5
PFBS	3%	<LOQ	<LOQ	n.d.-0.14	20%	0.034	<LOQ	n.d.-0.24
PFHxS	0%				93%	1.1	0.90	n.d.-3.9
PFOS	73%	0.68	0.43	<LOQ-3.3	100%	3.5	1.9	0.62–16
PFOS-br	73%	0.52	0.30	n.d.-2.9	100%	1.0	0.70	0.28–2.9

n.d., not detected; <LOQ, below the limit of quantification.

2,4-DBP was detected at the highest level, with a median concentration of 200 ng/g saliva and 340 ng/g serum. A background contamination (53 ng/g sample, RSD 27%) was identified, likely originating from the sample pretreatment (method blank, Supplementary Table S3). Levels found in the saliva sampling QC sample were linked to the sample pretreatment and not to the sampling equipment. Unfortunately, no dog serum sampling blank was available in this study. In the previously published method (7), the sampling blank for 2,4-DBP analyzing human serum was below the method blank, which supports the notion that the contamination originated from the chemicals used in the pre-treatment method and not during sampling. Instead, the EMR powder was previously reported to be the primary source of 2,4-DBP contamination in the validated method, despite washing the EMR powder twice with distilled ACN (7).

The two approved feed additives, BHA and BHT, could be found in all dogs. BHA levels were similar in saliva and serum samples (median 1.8–1.9 ng/g and range n.d.-12 ng/g). BHT were primarily found as its metabolites, BHT-CHO, 3,5-di-tert-butyl-4-hydroxybenzoic acid (BHT-COOH), BHT-OH, BHT-Q, and 2,6-di-tert-butyl-4-hydroxy-4-methyl-2,5-cyclohexadienone (BHT-quinol). The median levels were below LOQ, but average levels of octyl 3-(3,5-ditert-butyl-4-hydroxyphenyl) propanoate (AO1135) were similar in saliva and serum (9–11 ng/g) and could be quantified in 31% of the saliva samples (range n.d.-140 ng/g saliva) and 48% of the serum samples (n.d.-87 ng/g serum).

Eight PFAS congeners were quantified in dog’s saliva and/or serum (Table 1). Primarily, PFNA (n.d.-0.82 ng/g saliva) and PFOS (linear and branched, <LOQ − 3.3 and n.d.-2.9, respectively) could be quantified in dog’s saliva, in 93 and 73% of the saliva samples, respectively. PFBS was quantified in only one saliva sample (0.14 ng/g saliva). In dog blood serum, all eight PFAS could be quantified in >20% of the samples, at levels of up to 19 ng of PFOS (linear and branched)/g of serum.

**Figure 1:**
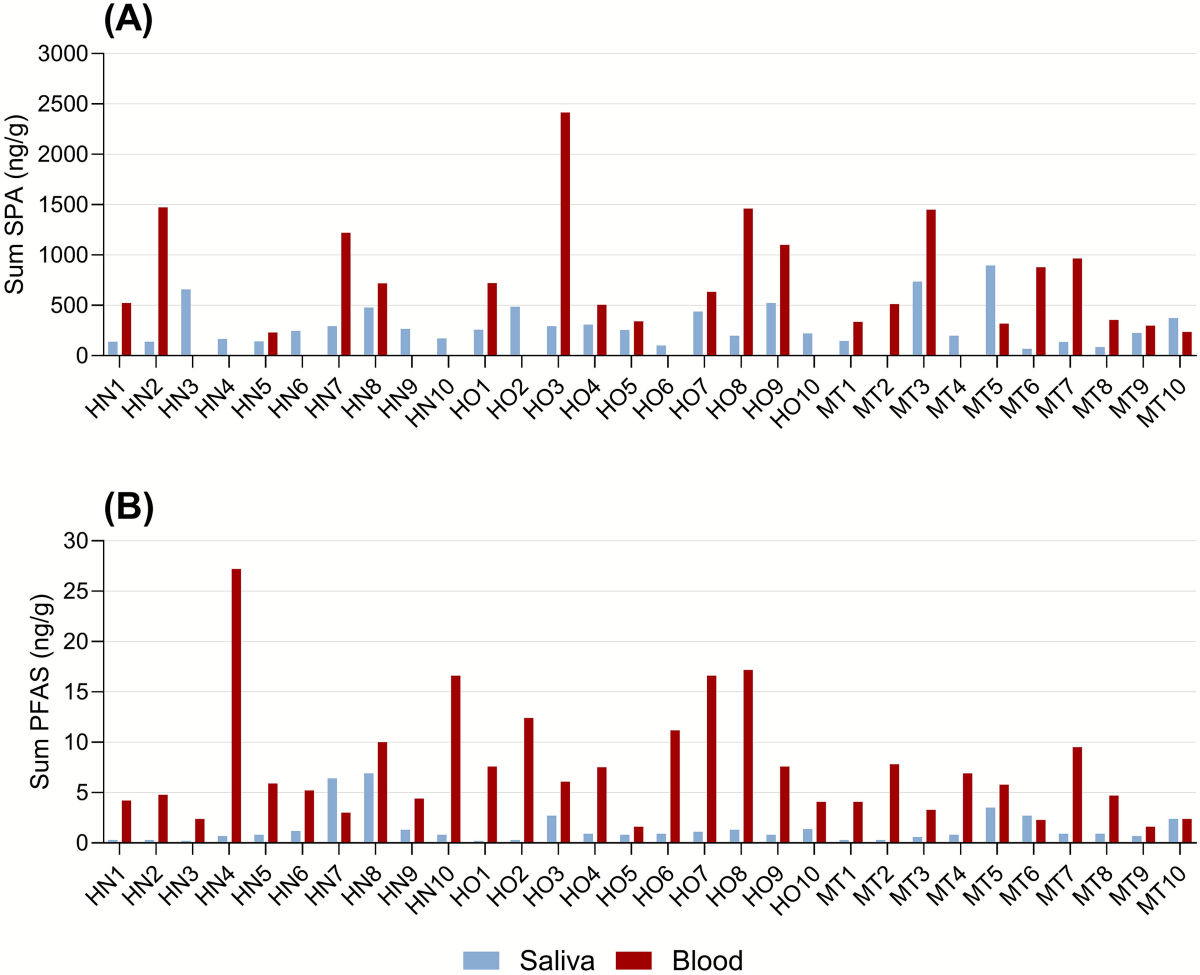
Sum of the SPA **(A)** and PFAS **(B)** levels (ng/g) determined in individual dog saliva (blue) and blood serum (red) samples. Observe that results of samples with IS recovery <30% for SPA determination are removed (Supplementary Table S3).

In Figure 1, all SPAs (*n* = 14, Figure 1A) and all PFAS (*n* = 8, Figure 1B) are summed to compare the total exposure in dog saliva and blood serum of each individual dog. The major SPA contributor to sum SPA was 2,4-DBP, constituting, on average, 83% in saliva and 72% in blood serum. The major PFAS contributor to sum PFAS was PFOS (linear and branched), constituting on an average 62% in saliva and 57% in blood serum.

### 3.3 Correlations and group comparison

Only analytes quantified in more than 30% of the samples were further evaluated statistically, resulting in 7 SPAs and 3 PFAS in saliva and 10 SPAs and 7 PFAS in blood serum (Supplementary Table S5).

**Figure 2:**
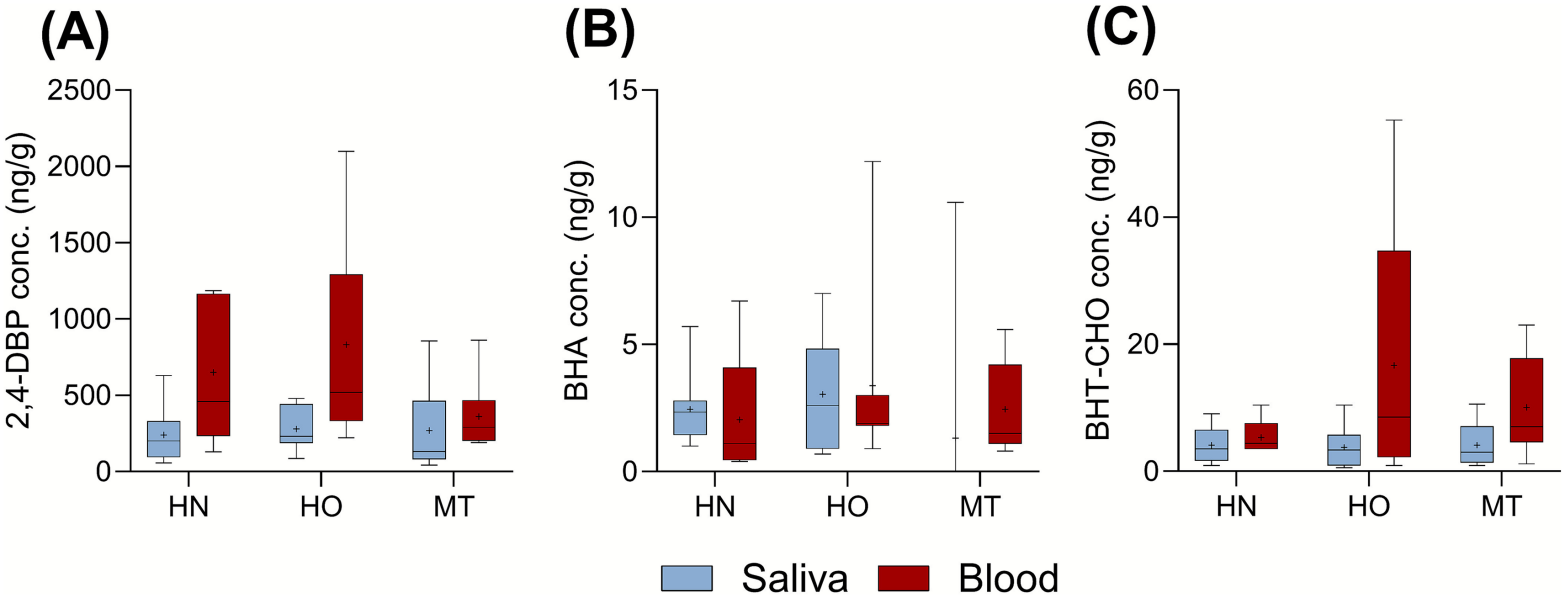
Concentrations (ng/g) of **(A)** 2,4-DBP, **(B)** BHA, and **(C)** BHT-CHO, quantified (QF) in >70% of both matrices. Saliva (blue) and blood serum (red). The results are divided on the health-status: healthy normal-weight (HN), healthy obese (HO), and dogs diagnosed with mammary tumour (MT). The box and whisker plot shows (from the top to the bottom) the max, 75^th^ quartile, median, 25^th^ quartile and min. The average is indicated with a plus.

**Figure 3:**
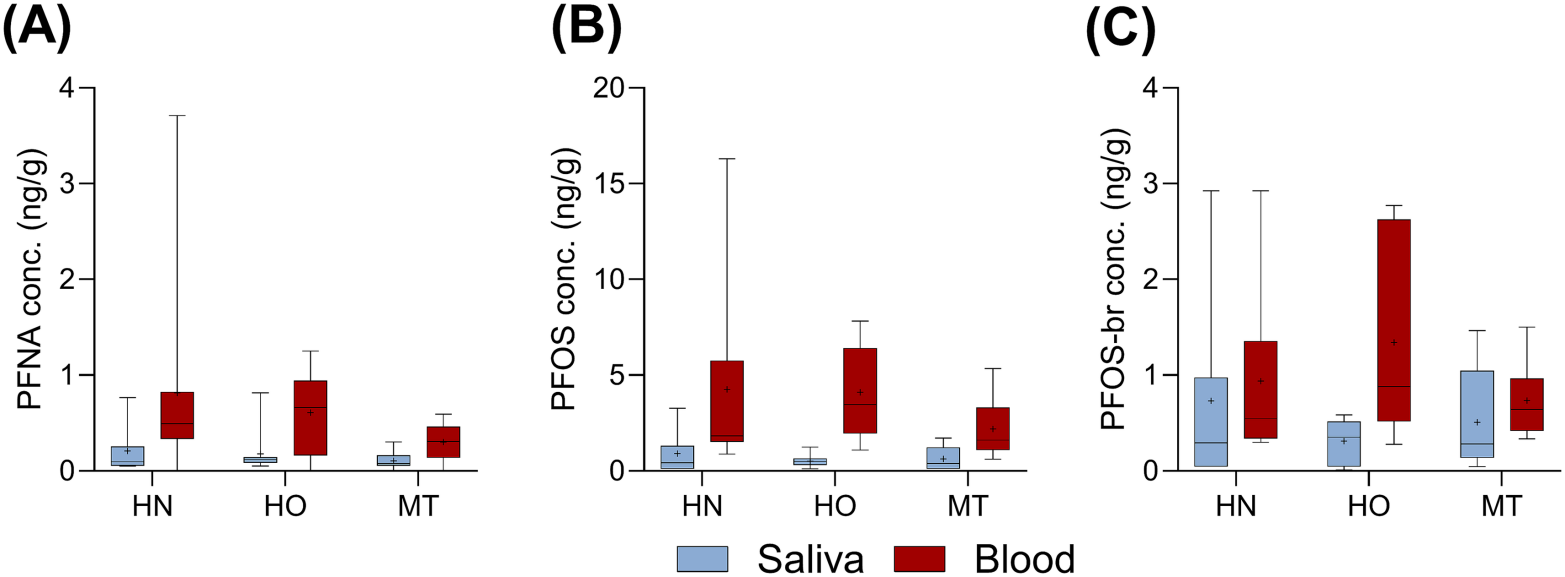
Concentrations (ng/g) of **(A)** PFNA, **(B)** PFOS-lin, and **(C)** PFOS-br quantified (QF) in >70% of both matrices. Saliva (blue) and blood serum (red). The results are divided on the health-status groups: healthy normal-weight (HN), healthy obese (HO), and dogs diagnosed with mammary tumour (MT). The box and whisker plot shows (from the top to the bottom) the max, 75^th^ quartile, median, 25^th^ quartile, and min. The average is indicated with a plus.

There were no correlations between any of the analytes in the paired saliva and blood samples. There were no statistically (Kruskal–Wallis) significant differences between the dogs with the three different health status; healthy normal-weight (HN), healthy obese (HO), and dogs with mammary tumours (MT) (Figures 2, 3 showing the analytes quantified in >70% in both matrices). In addition, sex, age, weight, and body condition score did not correlate to the target analytes.

The analytes were tested for correlation within the two matrices (Supplementary Table S5). In saliva, 2,4-DBP correlates positively with BHT-CHO (*r* = 0.448, *p* = 0.015) and PFNA (*r* = 0.613, *p* = 0.000) but negatively to BHT-Q (*r* = −0.391, *p* = 0.04). AO1135 correlates negatively to PFOS (*r* = −0.441, *p* = 0.017). In serum, BHT-COOH correlates positively to AO1135 (*r* = 0.561, *p* = 0.008). BHT-Q correlates positively to 2,4-DBP (*r* = 0.452, *p* = 0.041), and BHA (*r* = 0.518, *p* = 0.017) but negatively to 2,4,6-tris-tert-butylphenol (AO246. *r* = −0.595, *p* = 0.004). BHT-quinol correlates negatively to 4,4′-methylenebis(2,6-di-tert-butylphenol) (AO4426. *r* = −0.489, *p* = 0.024), BHT-OH (*r* = −0.449, *p* = 0.041), but positively to BHT-Q (*r* = 0.532, *p* = 0.013). PFNA correlates positively to all PFAS (*r* = 0.442–0.69, *p* < 0.015).

## 4 Discussion

No blood or saliva samples from the 30 dogs in this study were free from the analysed contaminants (Figure 1). Thus, this study could confirm that SPAs and PFAS can be analysed in the saliva of dogs. There were fewer analytes detected in saliva (*n* = 10) than in blood serum (*n* = 17). One explanation could be the difference in physicochemical properties of the two groups, especially regarding the PFAS (Supplementary Table S2). The pH of both blood and saliva ranges approximately 7, which indicates that analysed PFAS are in their ionic forms, whereas all SPAs except BHT-COOH are in their neutral form.

In all serum and most saliva samples (72%), BHA could be quantified (Table 1). In humans, after a single BHA administration, its excretion was found to be primarily with urine (approximately 50% during the first day, and up to 80–90% during the following 11 days) and at a minor rate with faeces (up to 0.3% of the dose per day) (27). Yet, to the best of the authors’ knowledge, the possible excretion of BHA with saliva has not been investigated. Nevertheless, comparable concentrations of this compound in both biofluids observed in the present study warrant further research to clarify whether elevated salivary BHA reflects increased excretion from systemic circulation in dogs chronically exposed via feed, or whether BHA is additionally absorbed through the oral mucosa and subsequently secreted into saliva.

**Figure 4:**
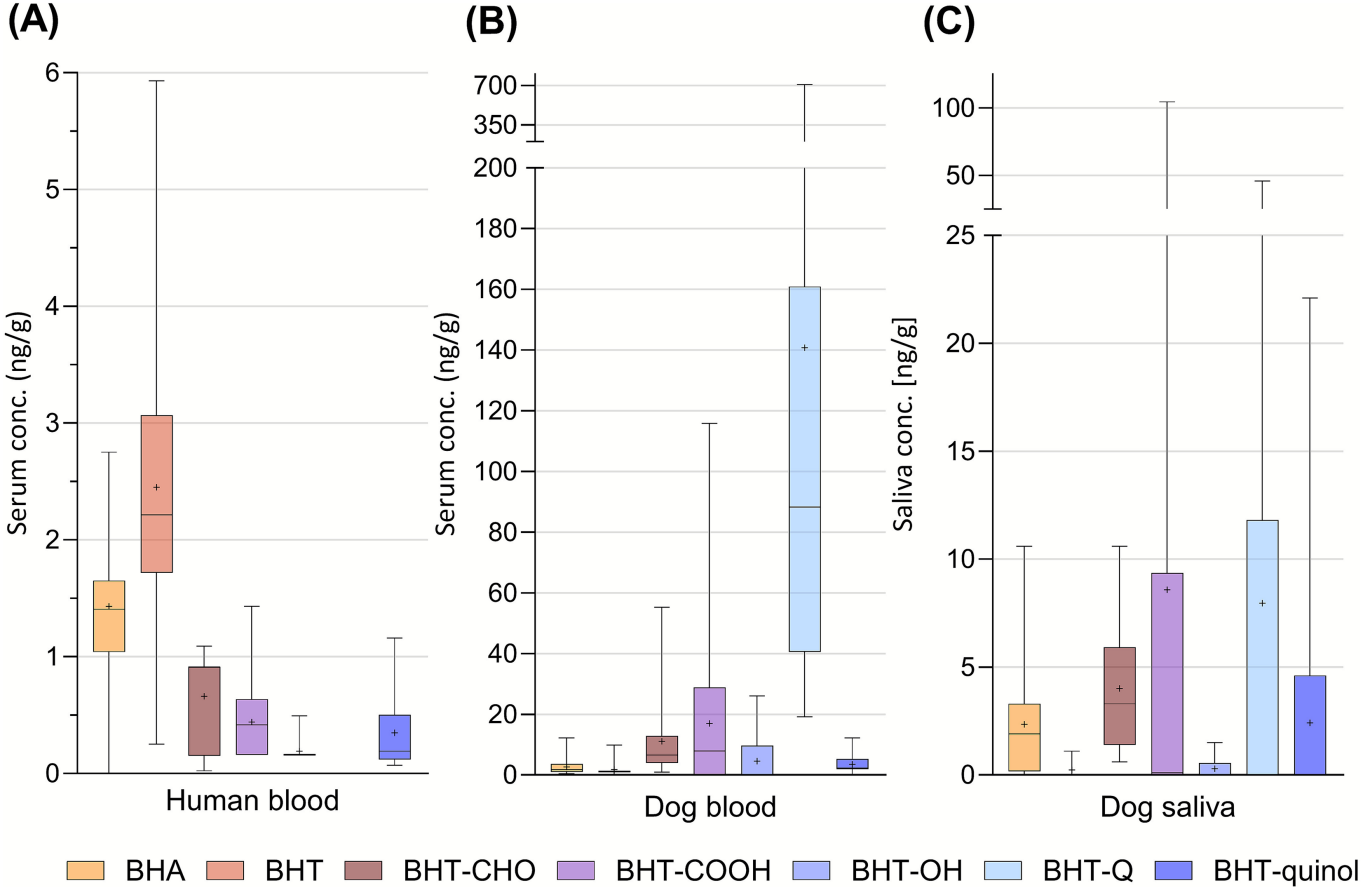
BHA, BHT, and the BHT-metabolites quantified (ng/g serum) in **(A)** human blood serum (only non-deconjugated fraction) (7) and in **(B)** dog blood serum, and **(C)** saliva. Observe that BHT-Q was not analysed in human serum. The box and whisker plot shows (from the top to the bottom) the max, 75^th^ quartile, median, 25^th^ quartile, and min. The average is indicated with a plus.

The BHA blood levels were almost twice as high in dogs (average 2.7, range 0.4–12 ng/g serum) in the present study compared to the reported blood serum levels in humans (average 1.5, range 0.7–2.8 ng/g serum) (7). The major metabolite of BHA, tert-butylhydroquinone (TBHQ) (28), could not be found in the dog’s serum or saliva in this study. In human serum, it was quantified in 37% of the samples (max 7.2 ng/g) of Swedish blood donors (7). On the contrary, while BHT could only be quantified in 14% of the dog’s serum samples (not in saliva), the BHT metabolites were found in most serum and saliva samples (Table 1). Compared to human serum, the relative presence of the BHT metabolites were more pronounced in the dogs (Figure 4). For example, the level of BHT-CHO was almost 50 times higher in dogs (average 11, range <LOQ-55 ng/g serum) compared to humans (average 0.2, range n.d.-1.1 ng/g serum). The high levels of BHT-metabolites could indicate a high exposure to BHT for dogs.

The BHT-metabolite BHT-Q (average 140, 19–710 ng/g serum in dogs, Figure 4) was not reported in the human serum samples due to background contamination issues (7). BHT-Q has been identified in FCM at a median concentration of 217 ng/g (29). In the serum of pregnant women, BHT-Q could be detected in 100% of the samples, and at a median level of 26.5 ng/mL serum (30). These findings suggest that exposure to SPAs may affect reproductive health, and it identifies BHT-Q as a potential biomarker for assessing early pregnancy loss risks.

In addition to exposure via feed and FCM, indoor companion animals are assumed to have a considerable intake of dust due to their grooming behaviour. BHT levels have been reported in household dust, ranging from 160 to 18,000 ng/g dust (median 1,600 ng/g dust) in China (14) and 2–200,000 (median 60,000 ng/g dust) in Sweden (31). Previous studies have demonstrated that both BHT and BHA can be oxidized under visible-light photoirradiation, although BHA has a lower oxidative potential compared to BHT (32). BHT metabolites, such as BHT-Q, BHT-CHO and BHT-quinol have been reported in household dust from China (14), at significant QF (>97% of the samples) and levels ranging between 18 and 4,500 ng/g of dust (median 460 ng/g dust). Although there are regional differences, dust intake can be a significant exposure pathway for the Spanish dogs analysed in this study, in addition to the added additives ingested with pet feed.

The high levels of 2,4-DBP in both blood serum (median 340 ng/g) and saliva (median 200 ng/g) could be considered as concerning (Figure 2), given the limited knowledge regarding its toxicity. It is important to stress that no blood sampling blank sample was analysed, and that background contamination from sample pretreatment corresponding to an average of 53 ng/g serum (RSD 27%) was established. Still, the high variation in the samples (<LOQ-2100 ng/g) confirms that 2,4-DBP is a high-production volume chemical, with widespread use. 2,4-DBP was identified to be the predominant SPA in FCM, such as disposable plastic cups and instant noodle buckets, with a median concentration of 886 ng/g FCM (29). Although a background contamination cannot be excluded, similarly high levels of 2,4-DBP have been reported in both human serum and urine (7, 9). Currently, 2,4-DBP is being evaluated under REACH for endocrine-disrupting properties (33).

By adding an enzymatic deconjugation step to the method, the conjugated fraction of SPAs in human serum could be determined (7). BHT-COOH and BHT-quinol levels increased, reaching up to 480 times higher than the free fraction alone (BHT-quinol: on average 29 times higher). The fraction of conjugated BHT-COOH was lower, with serum levels increasing by up to 2.3 times compared to the free fraction alone (on average 1.2 times). In this study, no deconjugation step was applied to determine the conjugated fraction. Hence, levels of these SPAs could be expected to be even higher than what is reported here.

AO1135 levels were similar in the dog’s saliva (average 9.3 ng/g saliva) and serum (average 11 ng/g serum). AO1135 could be found in 31% of the dog’s saliva and 48% of the dog’s serum, but have been reported below detection limit in human serum (7). On the contrary, AO2246 was found at high levels in human serum (QF 100%, average 520, range 260–830 ng/g serum) but not found in dogs. AO2246 has been reported in all (100% of *n* = 50) packed foodstuff samples in China, at an average level of 62.5 ng/g, which could explain the high levels in human serum (29).

AO4426 was found in both human (QF 93%, average 14, range <LOD-29 ng/g serum) and dogs (QF 76%, average 3.0, range n.d.-21 ng/g serum). AO4426 has been prioritized as a new and emerging risk chemical based on market data and the exposure index applied to the Swedish Product Register (34). AO4426 is being assessed for persistent, bioaccumulative, and toxic properties and evaluated for endocrine-disrupting properties (35).

**Figure 5:**
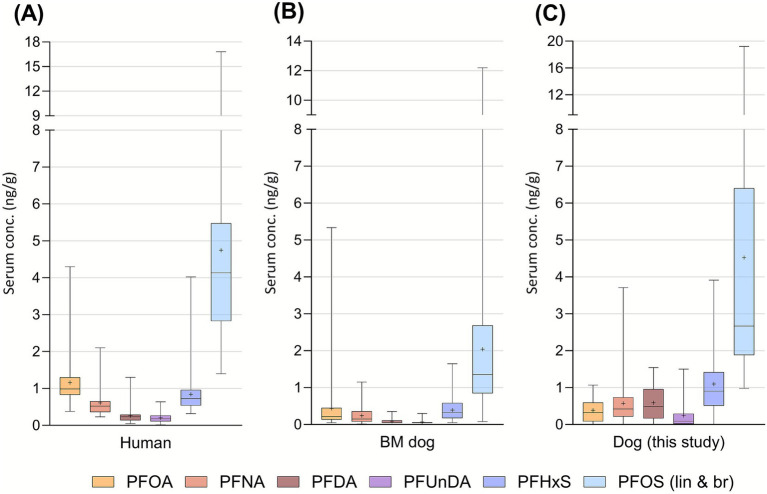
PFAS quantified (ng/g serum) in human blood serum (7), in Bernese Mountain (BM) dog blood serum (21) and dog blood serum (this study). The box and whisker plot shows (from the top to the bottom) the max, 75^th^ quartile, median, 25^th^ quartile, and min. The average is indicated with a plus.

Three PFAS were quantified in >70% of both the saliva and blood serum samples (Figure 3). No correlation between the two matrices could be established. The dog PFAS levels were similar to what was previously reported in a Swedish human population (Figure 5), using the same human blood samples as used for SPAs analysis (25). Only PFOA level was lower in dogs (average 0.4 ng/g serum, range n.d.-1.1) than in humans (average 1.2, range 0.4–4.2 ng/g serum). In a recent study, we reported PFAS levels in Bernese Mountain dogs from Sweden (21). The levels were in general lower than that reported in this study (Figure 5). To date, there are no data on PFAS levels in human saliva or in any other animal’s saliva to compare.

Dog exposure to PFAS is similar to human exposure. Both increased (PFNA and PFDA) and lowered (PFOA and PFOS) cholesterol levels with higher PFAS levels have been reported in beagles from China (36). To the best of the authors’ knowledge, typical health effects observed in human epidemiological studies have not been established in dogs, for example, lowered birth weight and immunotoxicity (22).

This study has some limitations. The relatively low sample size (*n* = 30) could have affected the ability to detect correlations due to individual variations. Confounding factors, such as breed, food intake, and other pathologies or environmental (e.g., rural versus cosmopolitan) conditions, have not been included here. Although efforts have been made to validate the analytical method, improvements in the sense of proper sampling blanks for blood serum, and recovery studies of the saliva as a new matrix are recommended. In addition, the sample volume of saliva was limited, and amount used for analysis ranged between 64 μL to 210 μL, with an average of 155 μL. Increasing the sample volume could improve the detection limits, and more analytes could potentially be quantified. Thus, this study should be considered as a pilot study, where a first screening was conducted.

### 4.1 Conclusion

This is the first time SPAs and PFAS levels have been reported in dog’s saliva. Despite limitations regarding sample size and method validation, this study confirms saliva to be a potential alternative non-invasive matrix to blood serum that motivates further investigation. It is recommended to verify these results in larger cohorts, as well as further studies on kinetics, using controlled exposure experiments.

Considering the lack of knowledge regarding the toxicity of the SPAS, the accumulated concentration in dog’s blood serum is concerningly high, reaching levels above 4 μg/g serum. As AO4426 and 2,4-DBP are currently under assessment as endocrine disruptors further motivates investigations of these high-production volume chemicals.

No correlations between analyte levels and health status could be statistically determined, which was expected due to the limited samples size. The comparative exposure assessment showed that humans and dogs are exposed to similar levels and that using dogs as sentinels for human exposure is a promising strategy. Comparative and translational studies on dogs as model organisms for endocrine disease, is a non-experimental animal study design within One Health and 3R.

## Data Availability

The original contributions presented in the study are included in the article/Supplementary material, further inquiries can be directed to the corresponding author.
